# Direct and Indirect Targets of the E2A-PBX1 Leukemia-Specific Fusion Protein

**DOI:** 10.1371/journal.pone.0087602

**Published:** 2014-02-04

**Authors:** Christofer Diakos, Yuanyuan Xiao, Shichun Zheng, Leo Kager, Michael Dworzak, Joseph L. Wiemels

**Affiliations:** 1 Department of Epidemiology and Biostatistics, University of California San Francisco, San Francisco, California, United States of America; 2 Department of Pediatrics, St Anna Children’s Hospital, Medical University of Vienna, Vienna, Austria; 3 Department of Neurosurgery, University of California San Francisco, San Francisco, California, United States of America; 4 Children’s Cancer Research Institute, St Anna Kinderkrebsforschung, Vienna, Austria; B.C. Cancer Agency, Canada

## Abstract

*E2A-PBX1* is expressed as a result of the t(1;19) chromosomal translocation in nearly 5% of cases of childhood acute lymphoblastic leukemia. The E2A-PBX1 chimeric transcription factor contains the N-terminal transactivation domain of E2A (TCF3) fused to the C-terminal DNA-binding homeodomain of PBX1. While there is no doubt of its oncogenic potential, the mechanisms of E2A-PBX1-mediated pre-B cell transformation and the nature of direct E2A-PBX1 target genes and pathways remain largely unknown. Herein we used chromatin immunoprecipitation assays (ChIP-chip) to identify direct targets of E2A-PBX1, and we used gene expression arrays of siRNA E2A-PBX1-silenced cells to evaluate changes in expression induced by the fusion protein. Combined ChIP-chip and expression data analysis gave rise to direct and functional targets of E2A-PBX1. Further we observe that the set of ChIP-chip identified E2A-PBX1 targets show a collective down-regulation trend in the E2A-PBX1 silenced samples compared to controls suggesting an activating role of this fusion transcription factor. Our data suggest that the expression of the E2A-PBX1 fusion gene interferes with key regulatory pathways and functions of hematopoietic biology. Among these are members of the WNT and apoptosis/cell cycle control pathways, and thus may comprise an essential driving force for the propagation and maintenance of the leukemic phenotype. These findings may also provide evidence of potentially attractive therapeutic targets.

## Introduction

E2A-PBX1 is expressed as a result of the t(1;19) chromosomal translocation in nearly 5% of cases of acute lymphoblastic leukemia. The E2A-PBX1 chimeric transcription factor contains the N-terminal transactivation domain of E2A fused to the C-terminal DNA-binding homeodomain of PBX1. Previews studies indicate that additional genetic events may be required for E2A-PBX1-leukemic transformation, based on long incubation times and monoclonal nature of tumors observed in mouse models [1]–[3]. While there is no doubt of its oncogenic potential, the mechanisms of E2A-PBX1-mediated pre-B cell transformation and the nature of direct E2A-PBX1 target genes and additional events that complement the fusion oncogene to create full-blown leukemia are still unclear. Current evidence supports the idea that the oncogenic activity of E2A-PBX1 is propagated by the aberrant activation of genes normally controlled by PBX/HOX complexes. PBX1 itself shows little or no transactivation potential. However, when it is part of the E2A-PBX1 translocation product, PBX1 has strong transactivation properties. These data derive mainly from mechanistic models using artificial promoters containing the PBX1 binding site, deletion mutants of E2A-PBX1 and reporter gene assays [1], [4]–[8]. There are also data identifying singular E2A-PBX1 downstream target genes like Wnt16 [9]–[11]. Nevertheless the spectrum of these E2A-PBX1 deregulated target genes in leukemia and the pathways that are engaged through the occurrence of this translocation are still elusive. We employed chromatin immunoprecipitation (ChIP-chip) assays to identify direct targets E2A-PBX1, and we used gene and miRNA expression arrays of siRNA E2A-PBX1-silenced cells to evaluate changes in expression induced by the fusion protein. We identify 108 direct E2A-PBX1 targets; the fact that the majority of these targets decreased expression upon siRNA silencing of E2A-PBX1 corroborates the activation potential of this translocation and enlightens its action on a global cellular stage. Pathway analyses of the of E2A-PBX1 direct and functional targets support the WNT pathway as a downstream target of this fusion gene product and present novel insights, including pathways involved in cell cycle control and apoptosis.

## Materials and Methods

The B-cell precursor leukemic cell line 697 (having the *E2A-PBX1* fusion gene was grown in 24-well plates (Nunc, Roskilde, Denmark) at 10^6^ cells/mL. SiRNAs targeting the fusion region of *E2A-PBX1* and controls were designed and synthesized by Dharmacon (Lafayette, CO). siRNA duplexes were handled essentially as described [12]. The transfection of 697 cells (German Collection of Microorganisms and cell cultures, DSMZ no.: ACC 42) with siRNA duplexes at a concentration of 230 nM was performed using Lipofectamin 2000 (Invitrogen, Carlsbad, CA) according to the manufacturer’s instructions and as described elsewhere [12]. Total RNA from *E2A-PBX1* siRNA-treated and control treated 697 cells was isolated using the RNeasy Mini Kit RNA isolation kit (QIAGEN, Valencia, CA). Further processing of RNA and hybridization to microarrays was performed by Roche Nimblegen (Roche Nimblegen, Madison WI). Preparation of cell lysates and Western blot analysis were performed as described previously [9], [12]. In brief, protein extracts were separated using mini gels (Invitrogen, Carlsbad CA) and analyzed by immunoblot analysis using a 1∶1,000 dilution of primary antibody and a 1∶10,000 dilution of a horseradish-peroxidase conjugated secondary antibody or 1∶1,000 AP-conjugated from Cell Signaling. Immunocomplexes were visualized with the enhanced chemiluminescence (ECL, Amersham Pharmacia) system or CDP-star (New England Biolabs). For chromatin immunoprecipitation, cells were harvested and chromatin was formalin crosslinked and sheared to approximately 200–1000 bp using a Sonic Dismembrator model 550 at settings 1.5, for 2–4 cycles 50 s per cycle on ice. We followed the microarray application protocol as described in the kit (EZ-ChIP Kit) to isolate chromatin DNA complexes (Upstate cat no 17–371). ChIP antibodies to *E2A-PBX1* (Becton Dickinson) and anti-RNA Polymerase II (Upstate, Lake Placid, NY) were used. Chromatin was also purified from crosslinked DNA that had not been immunoprecipitated; this served as an input DNA control for the arrays, which was co-hybridized to provide a reference (that is, total input amplicon). To prepare samples for array hybridization, we applied the method developed in Bing Ren’s laboratory to carry out ligation-mediated PCR amplification of ChIP DNA [13]–[15]. At least 4 μg of ligation-mediated PCR-amplified DNA was used to carry out the subsequent labeling and hybridization steps. The IP amplicon was labeled with Cy5, and an equal amount of the total input amplicons labeled with Cy3; the two are combined to hybridize to the human promoter array (Roche Nimblegen, Madison WI). The design of the “2006-07-18_HG18_RefSeq_promoter” array is based only on normal RefSeq genes (NM_) for human (HG18; NCBI Build 36). The array includes 2200 bp upstream of transcriptional start and 500 bp downstream. Isothermal probe design uses dual tm/cycle restriction: 50–75 bp, tm 76, 148 cycles, 15-mer frequency of 50. Interval spacing of the probes is 100 bp. The experiment was performed in triplicate. For array CGH experiments, patients were derived from the Northern California Childhood Cancer Study, an epidemiology study, and from the Children’s Oncology Group DNA bank. Conventional karyotyping identified all patients as t(1;19), and molecular cloning of the translocation provided confirmation [16]. All patient’s parents provided informed consent, and the research was reviewed by the Institutional Review Board at UCSF. Mononuclear cells were isolated from diagnostic bone marrow. DNA was isolated using conventional SDS-proteinase K/ethanol precipitation methods. Array CGH was performed using *Nsp*1 Affymetrix arrays, which type approximately 280,000 single nucleotide polymorphisms. Labeling, hybridization, and scanning were performed using the Affymetrix product manual. Data were normalized using the global median method; medians for each array are estimated based on the 3,077 control probes “RANDOM_GC50_TM76”. The t “Max-four” approach by Krig et al. [17] was adopted. Data were first sorted according to genomic locations and a window of size *400 *bp moving along the genome, centering at each probe. For each probe, the median log-ratio of neighbor probes residing in the window was calculated and for each promoter, the highest smoothed value of the probes located within the promoter region was assigned as its “Max400” score. We obtained the genes that have top 500 Max400 scores in at least 2 of the three arrays. Expression arrays of three E2A-PBX1 silenced samples and three controls were performed. Data were normalized using quantile normalization. Correlation among the 6 arrays was then examined. A correlation plot of the three samples using log-ratios formed by subtracting mean of the 3 controls for each gene was generated quantile normalization (Figure S1A and S1B). Based on the above pairs plot, one sample was removed and the 2 remaining samples and 3 controls were used for analysis. Differential expression analysis gave rise to 122 significant differentially expressed genes between E2A-PBX1 silenced samples and controls (Table S1 and Table S2 show the top 30 up and down regulated). The log_2_ ratios of mRNA promoter pull-downs were graphed against the log_2_ ratios of the mRNA expression arrays. KEGG Pathway analysis of direct and functional E2A-PBX1 targets was performed using Exploratory Gene Association Networks (EGAN) software [18] and direct and functional targets and pathways that might be regulated by them were visualized. Array data for this experiment is uploaded to the gene Expression Omnibus at reference number GSE52655.

### Ethics Statement

The work described was reviewed and approved by the Committee on Human Research (CHR), which is UCSF’s Institutional Review Board. The principles expressed in the Declaration of Helsinki were utilized in this research. The participants or their parents/guardian (if a minor) provided written consent to participate in this study.

## Results and Discussion

**Figure 1:**
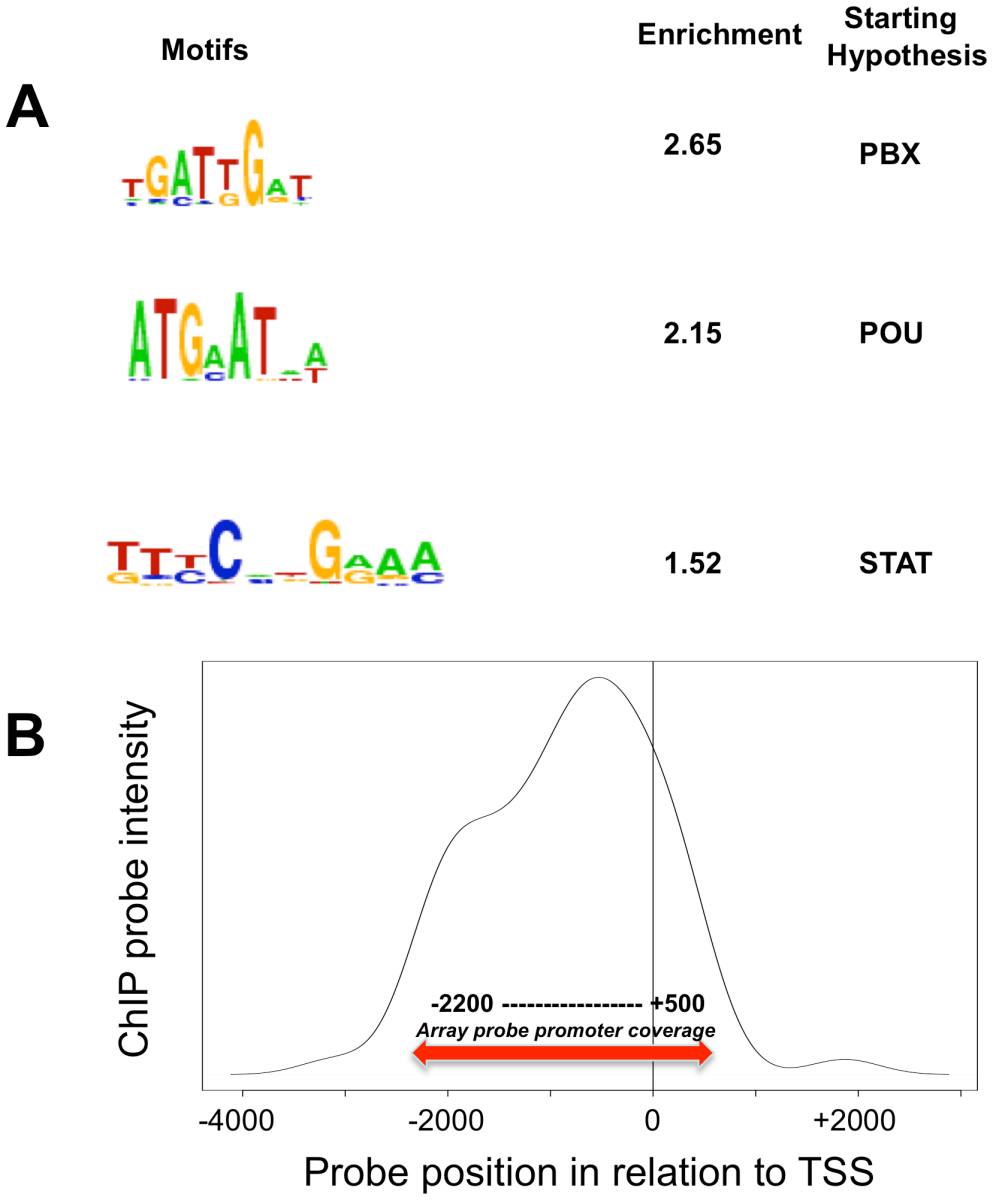
E2A-PBX1 ChIP binding. **A. Peak promoter sequence analysis**. Chromatin immunoprecipitation assays were performed to identify direct targets E2A-PBX1 using the human promoter array; HG18; (NCBI Build 36, Roche Nimblegen, Madison WI). A search was performed using the software WebMOTIFS and de novo motif finding a search for Transcription Factors that are likely to contact and regulate the binding sites identified by ChIP-chip. The 3 most significant transcription factor families (classes of transcription factors most likely to regulate the input sequences) are PBX, POU and STAT. **B. Positional binding of E2A-PBX1**. Among the 108 top hit ChIP genes, 76 genes have a single annotated transcription start site. The average intensity of ChIP pull-down is graphed along the promoter region in a smoothed plot. This histogram of the positions of maximal peak intensities (mean of 3 replicates) relative to the transcription start site (TSS) in target genes (Table S3). Most target genes show a maximum probe intensity around −500 bp upstream of the TSS. Probe regions with multiple TSS were excluded from this analysis. The promoter coverage area was complete for all genes between −2200 and +500 by the TSS (solid line) with some additional coverage for some genes outside of that region (dotted line).

Transcription factors typically bind promoter regions and thus regulate the expression of their target genes. E2A-PBX1 is an important leukemogenic chimeric transcription factor and its targets have been addressed before; however, a genome wide promoter binding study has not been performed [1]–[3], [5], [6]. Using the leukemia cell line (697) expressing E2A-PBX1 and a specific antibody against E2A-PBX1, we performed chromatin ChIP-chip to identify mRNA and miRNA promoters bound by E2A-PBX1. We obtained the genes that have top 500 Max400 scores [17] in at least 2 of the three experiments which gave rise to 108 genes. For each of these 108 genes (Table S3), we obtained the location of the peak (highest smoothed value of probes within the same promoter region) for each array, and examined the correlations of these locations as well as for the rest of the genes (Figure S1A and S1B). We then performed peak probe sequence analysis requiring that the peak be found in the same location in at least two of the three independent experiments, giving rise to 75 peak sequences of length 50–65 nt. To search for common motifs, we searched for known transcription factor binding motifs that were significantly enriched in the 108 E2A-PBX1 ChIP-chip targets. The 3 most significant transcription factor families found were: PBX, POU and STAT (Figure 1A). The fact that the canonical PBX1 motif is highly enriched in our experimental ChIP pull down indicates the validity and robustness of our experiments. This PBX1 motif is similar to that found in previous studies that characterized a consensus motif for PBX1 (GTCAATTAAAGCATCAATCAATTTCG, note underlined section [19]). In addition the enrichment of POU and STAT binding sites might imply their potential involvement within E2A-PBX1 in transcription regulation directly or as cofactors of E2A-PBX1. Recently Huang et al. [12] show a comparative motif enrichment analysis with an increased level enrichment of PBX and STAT bound to a HOX gene family member. Importantly, the replacement of PBX1 with E2A-PBX1 in DNA-binding complexes containing HOX proteins has been shown to alter the regulation of HOX/PBX1 target genes [6], [20]. Notably, the strongest average binding within E2A-PBX1 promoters was approximately 500 bp prior to the transcription start site (Figure 1B).

**Figure 2:**
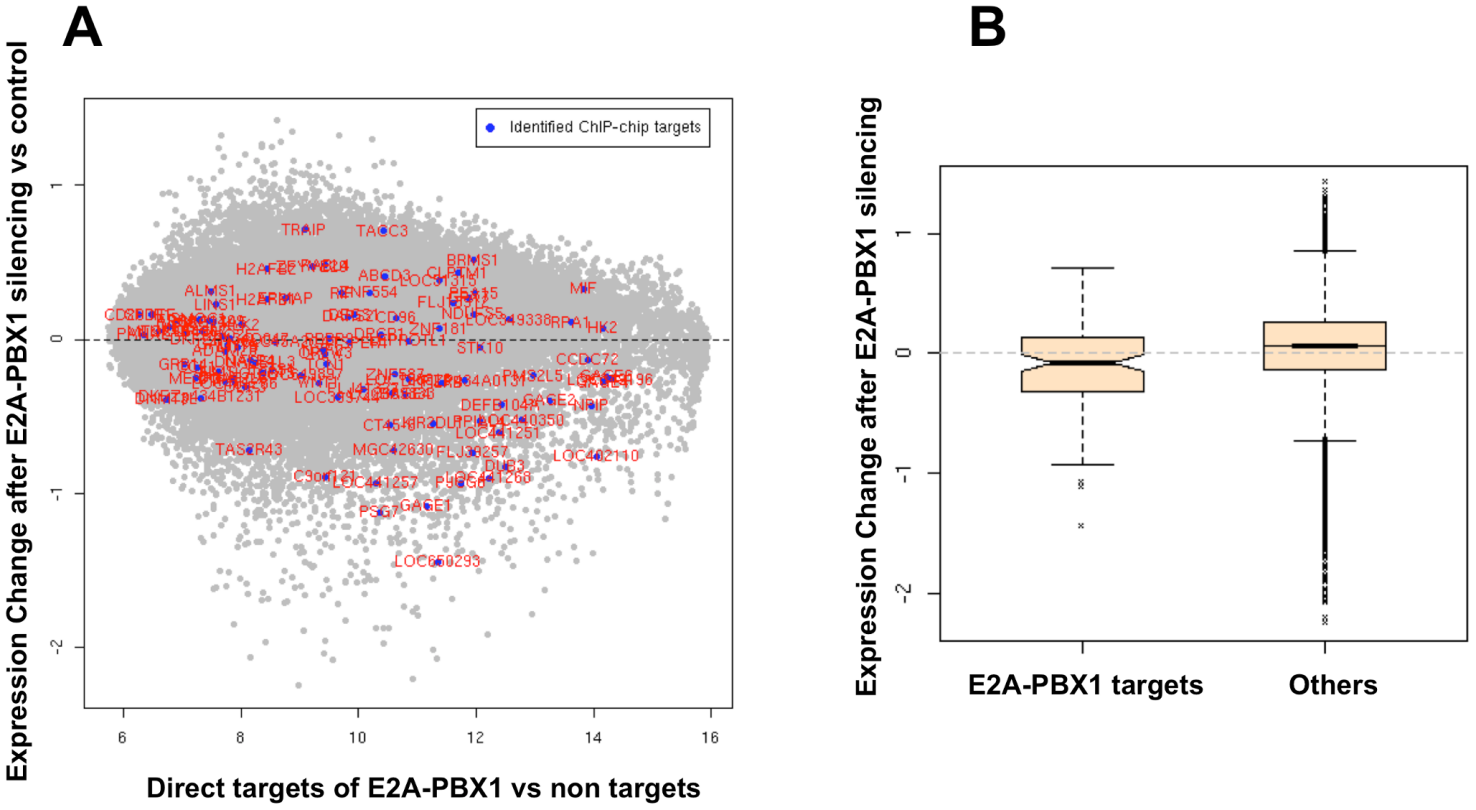
Identified ChIP-chip targets on expression array in E2A-PBX1 silenced vs control cells. **A.** ChIP-chip analysis data and expression analysis data upon E2A-PBX1 silencing were combined to get an overview of the direct and functional targets of E2A-PBX1. siRNA to E2A-PBX1 was employed to silence E2A-PBX1. The MA plot depicts the expression array changes upon silencing E2A-PBX1. Genes are ordered on the x-axis on the basis of their expression in untreated 697 cells. The Y-axis displays the change in expression upon E2A-PBX1 silencing. Those genes that are direct hits of E2A-PBX1 by chromatin immunoprecipitation assays (108 genes) were plotted against the expression array changes upon silencing and are shown in red color and marked with a blue dot; grey dots indicate all other genes assessed by the array. **B.** Here we compare the changes in expression of the 108 E2A-PBX1 direct targets to the expression change of all the other genes (non-direct E2A-PBX1 targets) upon E2A-PBX1 silencing. The set of identified ChIP-chip targets show collective down-regulation trend in the E2A-PBX1 silenced samples compared to controls (p<1.63e-06).

**Figure 3:**
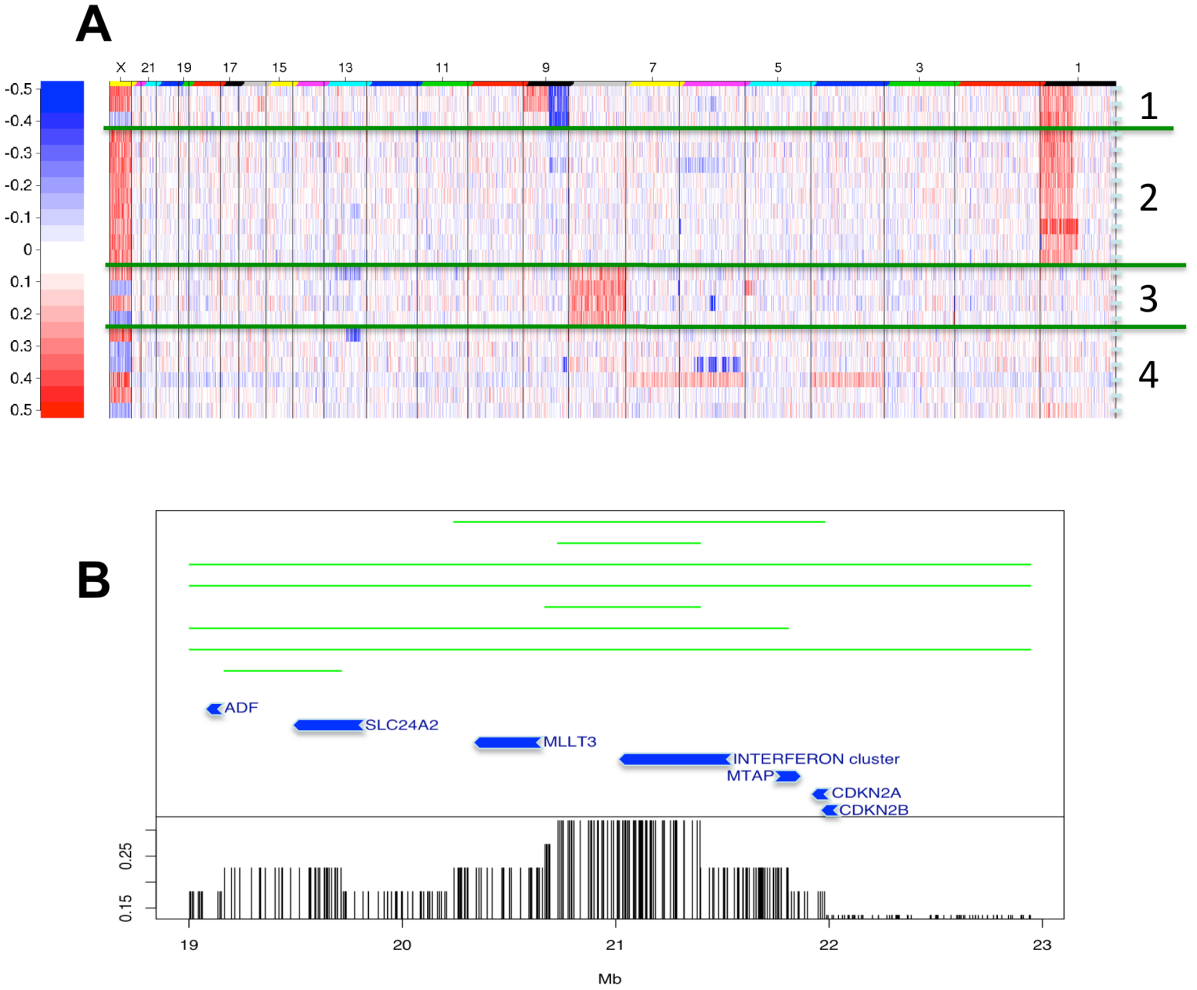
The 9p21 deletion is not always targeting CDKN2A. Copy number alterations in a group of 22 t(1;19)^+^ *E2A-PBX1* bone marrow samples are shown **Figure 3A**. Patient samples were subjected to hierarchical clustering analysis; we found the 9p21 deletion to exist in four of the 22 patients we have analyzed, with one patient exhibiting a homozygous deletion, white color refers to no changes, red to gain and blue to loss. **Figure 3B**. Fine map of deletions in the 9p21 region showing the deletion endpoints among 8 patients. The commonly deleted region among 10 patients (with 45%, including 7 patients in this figure and the other 3 others with complete arm loss) is the interferon gene cluster telomeric to the *CDKN2A/B* locus.

The E2A-PBX1 translocation results in the expression of an abnormal hybrid transcription factor capable of cellular transformation as shown in different of *in vitro* and *in vivo* assays [4]–[7]. We here investigated the impact of E2A-PBX1 expression upon mRNA expression to identify E2A-PBX1 dependent genes. siRNA was used to knock down E2A-PBX1 expression and expression arrays were used to study the impact upon gene expression. Silencing of E2A-PBX1 in 697 cells was performed as described by Casagrande *et al.* [9] and protein depletion was monitored by western blot analysis (Figure S2). Subsequently, total RNA was isolated and gene expression was performed using Nimblegen expression arrays. A display of the overall relationship of ChIP-binding and expression change after silencing *E2A-PBX1* is displayed in Figure 2A. A reduction in E2A-PBX1 protein resulted in a decreased expression of its target genes (Figure 2B). Upon E2A-PBX1 silencing expression analysis gave rise to 122 significant differentially expressed genes between E2A-PBX1 silenced samples and controls. The top 30 up and top 30 down regulated genes are shown in Table S1 and Table S2. The most profound change observed among these findings was the up-regulation of the cyclin-dependent kinase inhibitor 2A gene *CDKN2A*. *CDKN2A* encodes two distinct proteins, p16INK4A and p14ARF, translated in alternate reading frames; both of these proteins are involved in cell cycle regulation [12]. Importantly p14ARF has been shown to be down-regulated by E2A-PBX1 [21]. This puts forward a model were E2A-PBX1 affects cell cycle by down regulating the expression of *CDKN2A.* The two genes encoded by *CDKN2A* p16 and p14 are potent cell cycle regulators. p16 is an inhibitor of cyclin-dependent kinase and inhibits Rb phosphorylation while p14 activates TP53 via interaction with the MDM2 protein. Inactivation of CDKN2A is an important molecular event for the involvement of the leukemic phenotype and deletions of the *CDKN12A/2B* locus are common in childhood ALL patients. However previously data suggest that other mechanisms must contribute to CDKN2A/2B inactivation as they show these deletions to be rare among patients with *E2A-PBX1* translocations [22], [23]. We found this feature to exist in four of the 22 patients we have analyzed, with one patient exhibiting a homozygous deletion (Figure 3A). Moreover we show that the target of the 9p21 deletion is not always CDKN2A; instead we show that the interferon cluster next to it is the commonly deleted region (Figure 3B). Despite the infrequent deletions, suppression of CDKN2A/2B is undoubtedly an important feature of this subtype of leukemia. Herein we show that expression of CDKN2A is functionally dependent on the presence/expression of E2A-PBX1 as it was up regulated upon siRNA silencing of the E2A-PBX1. This might be achieved via suppression by the polycomb repressor protein, BMI1, which is specifically activated by E2A-PBX1 [21]. We found BMI1 expression to be down regulated upon siRNA silencing of the E2A-PBX1 providing further support for this E2A-PBX1-BMI1-CDKN2A connection. However BMI1 was not found to be a direct target of E2A-PBX1. In order to uncover whether direct E2A-PBX1 targets are functionally affected by E2A-PBX1 silencing we combined the expression data from the E2A-PBX1 siRNA silencing experiment together with the data from ChIP-chip analysis (Figure 2A). The set of identified ChIP-chip targets show collective down-regulation trend in the E2A-PBX1 silenced samples compared to controls Figure 2B. This finding is essentially very close to previous findings that suggest an activating role of the E2A-PBX1 protein (32–33), reinforcing the concept of the engagement of otherwise silent programs that might contribute to malignant transformation.

**Figure 4:**
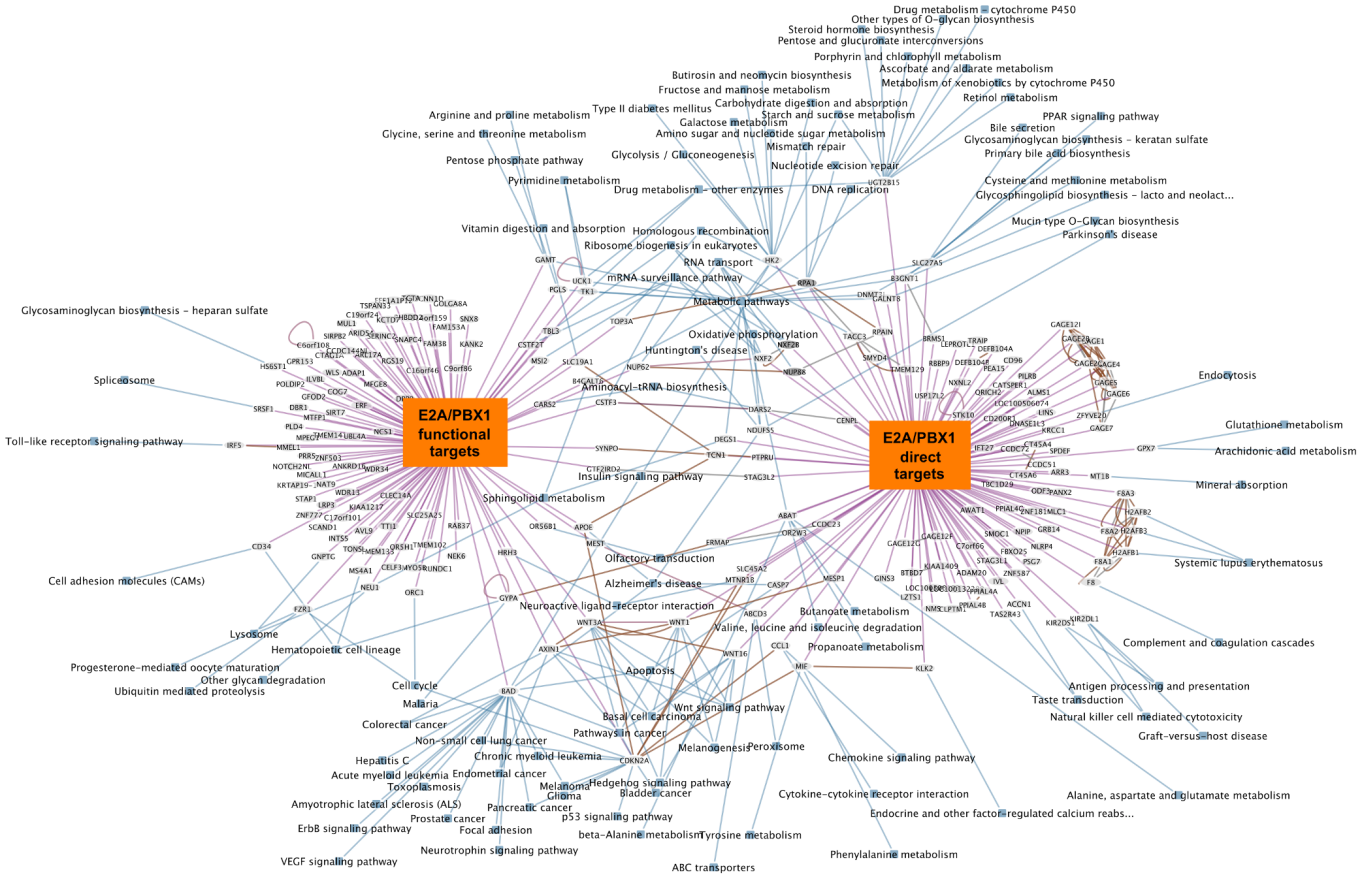
Combined KEGG Pathway analysis of direct and functional E2A-PBX1 targets. The analysis was performed using Exploratory Gene Association Networks (EGAN) software tool. E2A-PBX1 direct (108 direct targets identified by ChIP-chip) and functional targets (122 significant differentially expressed genes between E2a-Pbx1 silenced samples and controls) and Pathways that might be regulated by them were visualized. Interfaces of direct and functional E2A-PBX1 targets are depicted. Magenta lines depict the connection of the genes to the direct target and/or functional target group; blue lines show the participation of the genes in KEGG pathways and brown lines show known interaction between genes connected.

**Figure 5:**
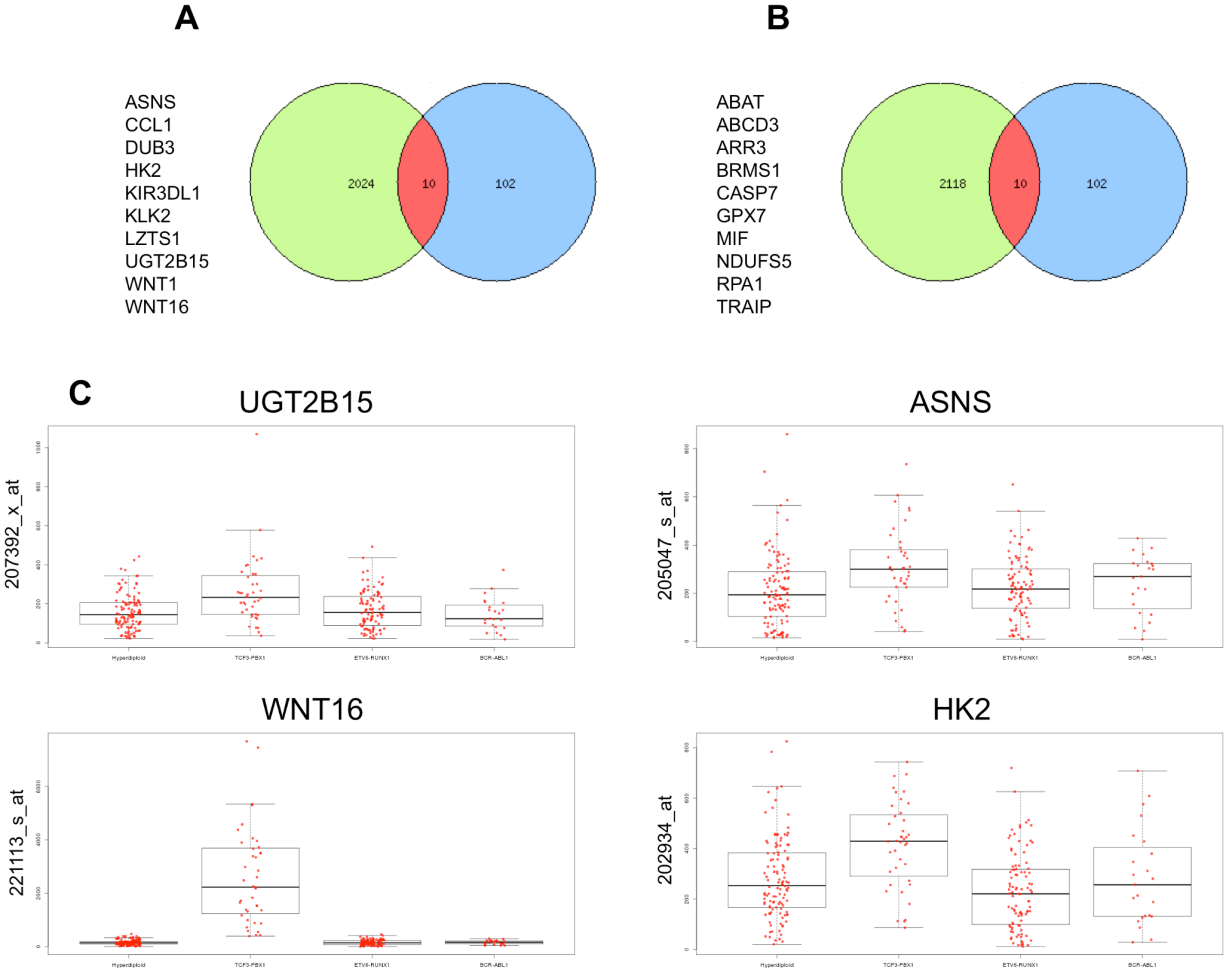
Expanded combined analysis of direct and functional E2A-PBX1 targets. The analysis was performed using the 102 direct targets identified by ChIP-chip analysis and the data set of the functional targets including the top 2000 genes up and down regulated after siRNA silencing of E2A-PBX1. Venn diagrams were generated and the interfaces of direct and functional E2A-PBX1 targets are depicted. 10 genes that were both direct targets and functionally down-regulated **(** **Figure 5A** **)** and 10 that were both direct targets and functionally up-regulated **(** **Figure 5B** **)** dependent on expression of E2A-PBX1 were identified. We are not able to address E2A-PBX1 as a functional repressor (for the 10 down regulated genes) or whether its depletion allowed accessibility to another transcriptional activator. **Figure 5C** Selected direct and functional E2A-PBX1 targets are highly expressed in primary E2A-PBX1 leukemias. Pediatric Cancer Genome Project. http://www.pediatriccancergenomeproject.org/site/accessed 14 Oct 2013. The expression of four genes UGT2B15, ASNS, WNT16 and HK2, that were among the direct and functional E2A-PBX1 targets in primary leukemia’s are shown. The expression of these genes was analyzed in Hyperdiploid leukemia’s (H), in E2A-PBX1 leukemia’s (E), TEL-AML1 (T) leukemia’s and BCR-ABL1 (B) leukemia’s are shown. The analysis of expression was performed using the gene expression tool and data available at St. Jude Children’s Research Hospital – (Washington University).

To explore the biological significance of the direct E2A-PBX1 target genes identified by ChIP-chip analysis we used the Exploratory Gene Association Networks (EGAN) software tool to visualize E2A-PBX1direct targets and pathways. KEGG Pathway analysis of 108 E2A-PBX1direct target genes (102 of which matched fully annotated genes) was performed and we could establish an involvement in the WNT pathway. The network of the E2A-PBX1 direct target genes and involved pathways is depicted on Figure S3. Further research will be necessary to unveil the complexity of the E2A-PBX1 bound/targeted promoters. However not only the direct targets of E2A-PBX1 are of interest, much more important is whether promoter binding translates into a functional modulation of the bound genes expression and whether downstream pathways and genes are affected. We therefore performed KEGG Pathway analysis of functional targets E2A-PBX1 that were differentially modulated upon siRNA silencing of E2A-PBX1. The analysis was performed using the Panther analysis online tool. Further the Exploratory Gene Association Networks (EGAN) software tool was used to visualize E2A-PBX1 functional targets and the Pathways that might be regulated by them. The pathways functionally affected by E2A-PBX1 included a spectrum of genes/pathways involved in major cellular processes (Table S4) such as leukemia signaling, apoptosis, control of hematopoetic lineage and cell cycle providing a global picture of the deregulated biology of the leukemic cell that is contributed by E2A-PBX1 (Figure S4). This comprehensive view includes the WNT pathway and cell cycle control genes as mentioned above as well as providing new insights. Further extensive research will be necessary to assess these new findings and address the complex connectivity between these E2A-PBX1 targets. Having produced two different data sets, ChIP-chip data with the direct targets of E2A-PBX1 and expression data of E2A-PBX1 silenced genes; we determined the commonality between these two data sets that might indicate both direct and functional targets. We found in these two data sets no genes that were both direct and functional targets. We further utilized the EGAN tool to find and visualize interfaces within the E2A-PBX1 direct and functional targets (Figure 4). In the center of this pathway network and in at the interface between the direct and functional E2A-PBX1 targets we find, among others, pathways involved in the regulation of the cell cycle, the regulation of apoptosis and importantly once again the WNT pathway. We were able to detect WNT16 as a direct target of E2A-PBX1 but not in the analyzed 122 functional targets data set as a direct target. However previous data has already shown that WNT16 was affected upon siRNA silencing of E2A-PBX1 [9], [11]. This and the fact that we initially were unable to find both direct and functional E2A-PBX1 targets with a limited cut point list prompted us to expand the data set of the functional targets including the top 2000 genes up and down regulated after siRNA silencing of E2A-PBX1. With this list, we were able to find 20 genes that were both direct targets and functionally dependent on expression of E2A-PBX1 (Figure 5A and 5B). Among these was WNT16. E2A-PBX1 has been already been shown to affect WNT16 expression as WNT16 expression was down regulated upon E2A-PBX1 silencing by siRNA [9], [11]. Herein we show WNT16 is a direct and functional target of E2A-PBX1 reconfirming and supplementing the findings of the previous studies. Analysis of WNT16 expression in primary leukemias shows that WNT16 is highly expressed in E2A-PBX1 leukemia’s compared to other pediatric leukemia subtypes, this was also true for other direct and functional targets of E2A-PBX1 (Figure 5C).

In addition we find another member WNT1 as a direct and functional target of E2A-PBX1 as it was down regulated upon silencing. This gene has been also implicated in leukemogenesis and WNT1 inhibition results in apoptosis of leukemic cells [9], [11]. Among the up regulated direct targets of E2A-PBX1 upon silencing we find, among others, CASP7 indicating an involvement in Apoptosis [24]–[26]. Silencing of E2A-PBX1 has been shown to result in apoptotic cell death that might at least in part be facilitated by the up-regulation of CASP7.

The E2A-PBX1 oncoprotein is generally a transcriptional activator. We used chromatin immunoprecipitation (ChIP-chip) assays to identify direct targets of E2A-PBX1 and we used gene expression arrays of siRNA E2A-PBX1 -silenced cells to evaluate changes in expression. We observe that the set of ChIP-chip identified E2A-PBX1 targets show practically a collective down-regulation trend in the E2A-PBX1 silenced samples compared to controls supporting an activating role of this fusion transcription factor. ChIP-chip and expression data analysis give rise to direct and functional targets of E2A-PBX1 and shows that the expression of the E2A-PBX1 fusion gene might interfere with key regulatory pathways and functions of leukemia biology. Among these members of the WNT and apoptosis/cell cycle control pathways, and thus may comprise an essential driving force for the propagation and maintenance of the leukemic process. Additional experiments in other model systems will be required to consider the totality of E2A-PBX1 mediated cell control our current report is largely limited to an observational link between E2A-PBX1 and targets. These findings may prompt further research and also provide first preliminary evidence of potentially attractive therapeutic targets.

## Supporting Information

Figure S1 **ChIP-chip Experimental design and experimental reproducibility.** The design of the “2006-07-18_HG18_RefSeq_promoter” array is based only on normal RefSeq genes (NM_) for human (HG18; NCBI Build 36). The array includes 2200 bp upstream of transcriptional start and 500 bp downstream. Isothermal probe design uses dual tm/cycle restriction: 50–75 bp, tm 76, 148 cycles, 15-mer frequency of 50. Interval spacing of the probes is 100 bp. This dataset is composed of 3 arrays of experiment vs 3 arrays control. The “Max-four” approach by King et al, JBC 2007 282:9703 was adopted. The genes that have top 500 Max400 scores in at least 2 of the three array experiments were adopted. This gave rise to 108 genes, 102 of which were annotated. For each of these 102 genes, we obtained the location of the peak (highest smoothed value of probes within the same promoter region) for each array experiment (experiment A, B and C) and examined the correlations of these locations among the top 102 genes (Figure 1a) and the rest of the genes (Figure 1b). The correlation in lower left side of side of the figure corresponds to the contra lateral data in the figure.
(PPTX)

Figure S2 **Western Blot Analysis.** Western blot analysis was performed to control E2A-BPX1 siRNA silencing for all 3 experiments prior to gene expression arrays. Control siRNA, siRNA targeting E2A-PBX1 and antibodies for E2A-PBX1 and tubulin detection were that were used as described previously by Casagrande et.al (Haematologica. 2006).
(PPTX)

Figure S3 **Pathway analysis of direct targets.** KEGG Pathway analysis of ChIP-chip E2A-PBX1 direct Targets was performed using Exploratory Gene Association Networks (EGAN) software tool. E2A-PBX1 direct targets and Pathways that might be regulated by them were visualized.
(PPTX)

Figure S4 **Pathway analysis of functional targets.** KEGG Pathway analysis of functional targets E2A-PBX1that were regulated upon siRNA silencing of E2A-PBX1 (Differential expression analysis of the top 122 significant differentially expressed genes between E2a-Pbx1 silenced samples and controls, both up and down regulated). The analysis was performed using Exploratory Gene Association Networks (EGAN) software tool. E2A-PBX1 functional targets and Pathways that might be regulated by them were visualized.
(PPTX)

Table S1 **Top 30 up regulated genes upon E2A-PBX1 silencing.** Top 30 Genes that were up-regaled upon E2A-PBX1 siRNA silencing are depicted 122 genes were significantly and differentially expressed genes between E2a-Pbx1 silenced samples and controls (up und down regulated genes).
(XLSX)

Table S2 **Top 30 down regulated genes upon E2A-PBX1 silencing.** Top 30 Genes that were down-regaled upon E2A-PBX1 siRNA silencing are depicted 122 genes were significantly and differentially expressed genes between E2a-Pbx1 silenced samples and controls (up und down regulated genes).
(XLSX)

Table S3 **E2A-PBX1 direct targets members of the WNT family.** ChIP-chip analyses of the E2A-PBX1 direct Targets are shown, among these, WNT16 and WNT1. The genes that have top 500 Max400 scores in at least 2 of the three arrays were adopted. This gave rise to 108 genes.
(XLSX)

Table S4 **Pathway analysis of functional targets E2A-PBX1.** Critical cellular pathways are regulated by E2A-PBX1. Panther Pathway analysis of functional targets E2A-PBX1 upon siRNA silencing was performed. Differential expression analysis of the top 122 significant differentially expressed genes between E2a-Pbx1 silenced samples and controls, both up and down regulated were used. The analysis was performed using Panther online gene analysis tool.
(XLSX)
