# Supplementary figures and images for: Direct and Indirect Targets of the E2A-PBX1 Leukemia-Specific Fusion Protein
Source: PLoS One. 2014 Feb 4;9(2):e87602. doi: 10.1371/journal.pone.0087602 (PMC3913655; doi:10.1371/journal.pone.0087602)

## Slide 1
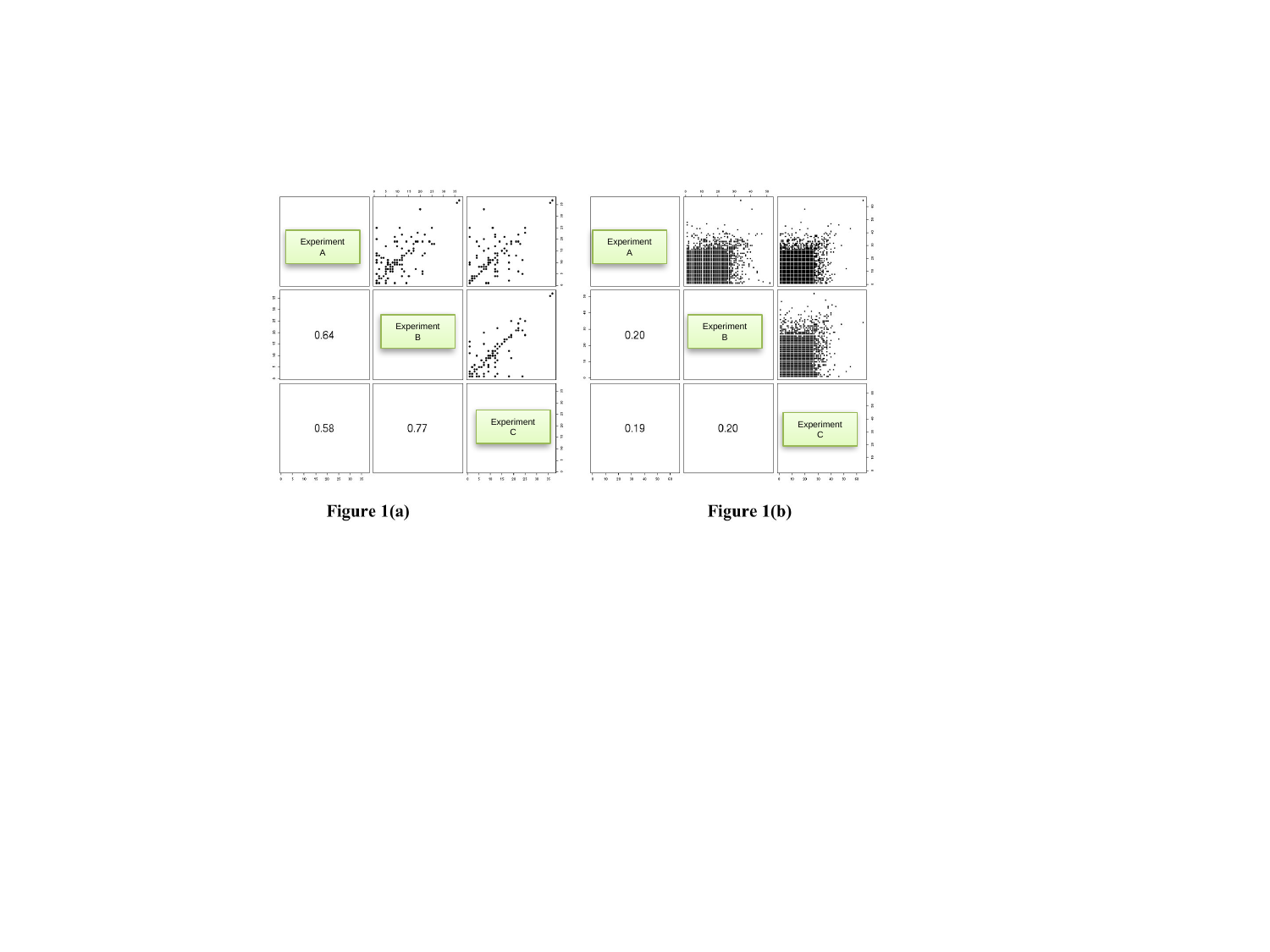

Experiment A
Experiment A
Experiment B
Experiment B
Experiment C
Experiment C

Supplement: Figure S1 — ChIP-chip Experimental design and experimental reproducibility. The design of the “2006-07-18_HG18_RefSeq_promoter” array is based only on normal RefSeq genes (NM_) for human (HG18; NCBI Build 36). The array includes 2200 bp upstream of transcriptional start and 500 bp downstream. Isothermal probe design uses dual tm/cycle restriction: 50–75 bp, tm 76, 148 cycles, 15-mer frequency of 50. Interval spacing of the probes is 100 bp. This dataset is composed of 3 arrays of experiment vs 3 arrays control. The “Max-four” approach by King et al, JBC 2007 282:9703 was adopted. The genes that have top 500 Max400 scores in at least 2 of the three array experiments were adopted. This gave rise to 108 genes, 102 of which were annotated. For each of these 102 genes, we obtained the location of the peak (highest smoothed value of probes within the same promoter region) for each array experiment (experiment A, B and C) and examined the correlations of these locations among the top 102 genes (Figure 1a) and the rest of the genes (Figure 1b). The correlation in lower left side of side of the figure corresponds to the contra lateral data in the figure. (PPTX) [file pone.0087602.s001.pptx]

## Slide 1
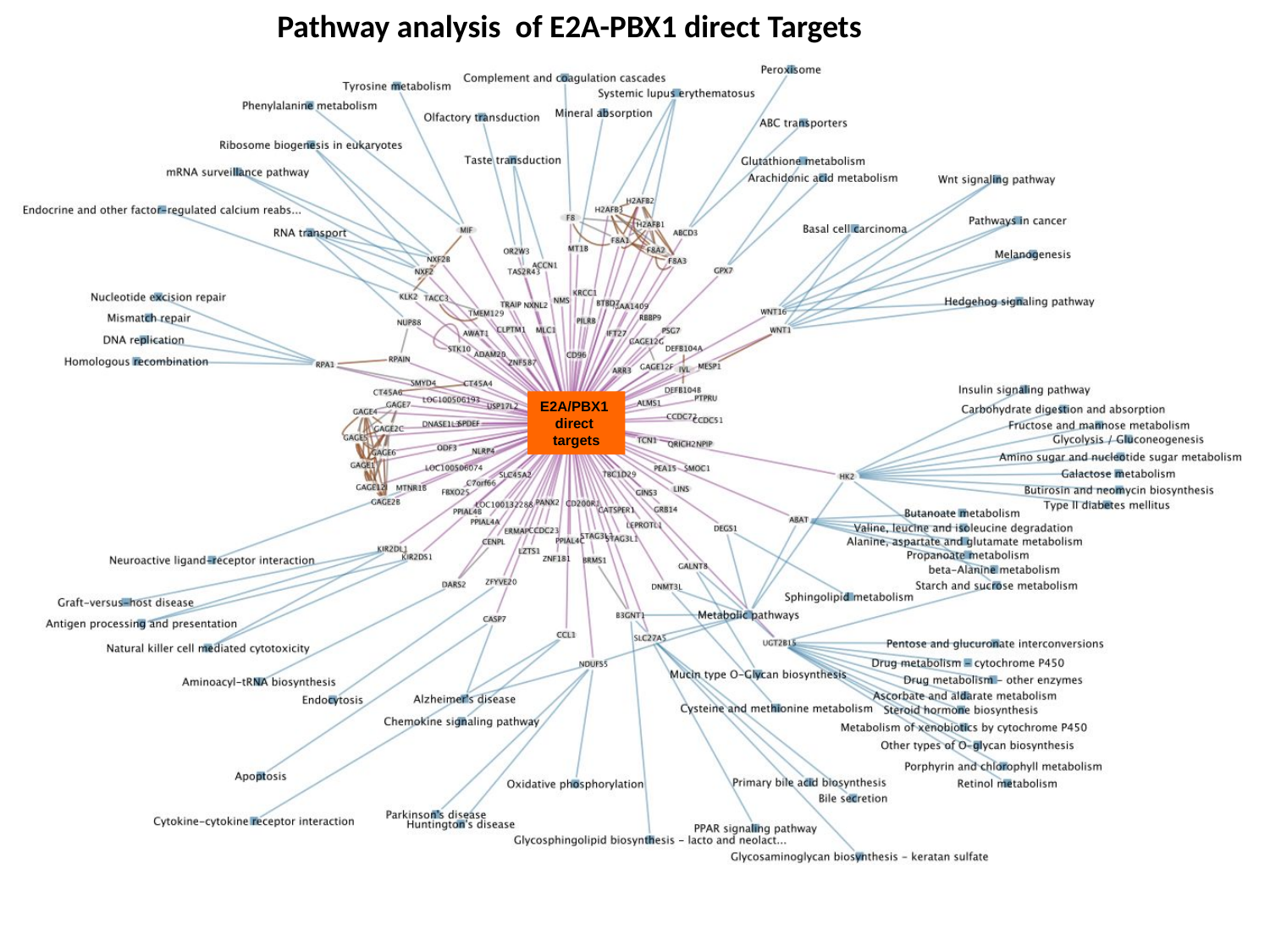

Pathway analysis of E2A-PBX1 direct Targets
E2A/PBX1
direct
targets

Supplement: Figure S3 — Pathway analysis of direct targets. KEGG Pathway analysis of ChIP-chip E2A-PBX1 direct Targets was performed using Exploratory Gene Association Networks (EGAN) software tool. E2A-PBX1 direct targets and Pathways that might be regulated by them were visualized. (PPTX) [file pone.0087602.s003.pptx]

## Slide 1
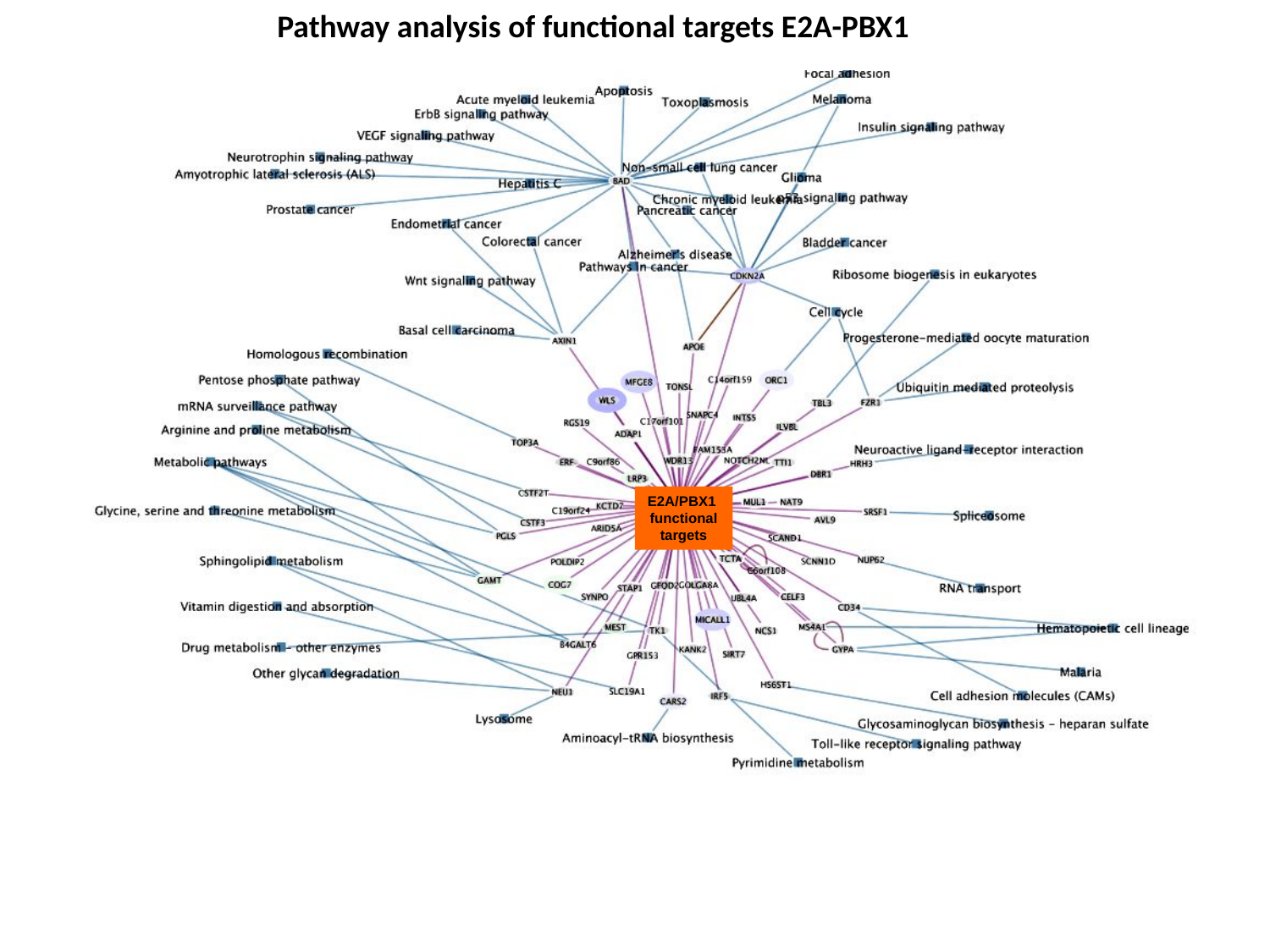

Pathway analysis of functional targets E2A-PBX1
E2A/PBX1
functional
targets

Supplement: Figure S4 — Pathway analysis of functional targets. KEGG Pathway analysis of functional targets E2A-PBX1that were regulated upon siRNA silencing of E2A-PBX1 (Differential expression analysis of the top 122 significant differentially expressed genes between E2a-Pbx1 silenced samples and controls, both up and down regulated). The analysis was performed using Exploratory Gene Association Networks (EGAN) software tool. E2A-PBX1 functional targets and Pathways that might be regulated by them were visualized. (PPTX) [file pone.0087602.s004.pptx]
