# Supplementary material for: Direct and Indirect Targets of the E2A-PBX1 Leukemia-Specific Fusion Protein
Source: PLoS One. 2014 Feb 4;9(2):e87602. doi: 10.1371/journal.pone.0087602 (PMC3913655; doi:10.1371/journal.pone.0087602)
Supplement: Figure S2 — Western Blot Analysis. Western blot analysis was performed to control E2A-BPX1 siRNA silencing for all 3 experiments prior to gene expression arrays. Control siRNA, siRNA targeting E2A-PBX1 and antibodies for E2A-PBX1 and tubulin detection were that were used as described previously by Casagrande et.al (Haematologica. 2006). (PPTX) [file pone.0087602.s002.pptx]

## Slide 1
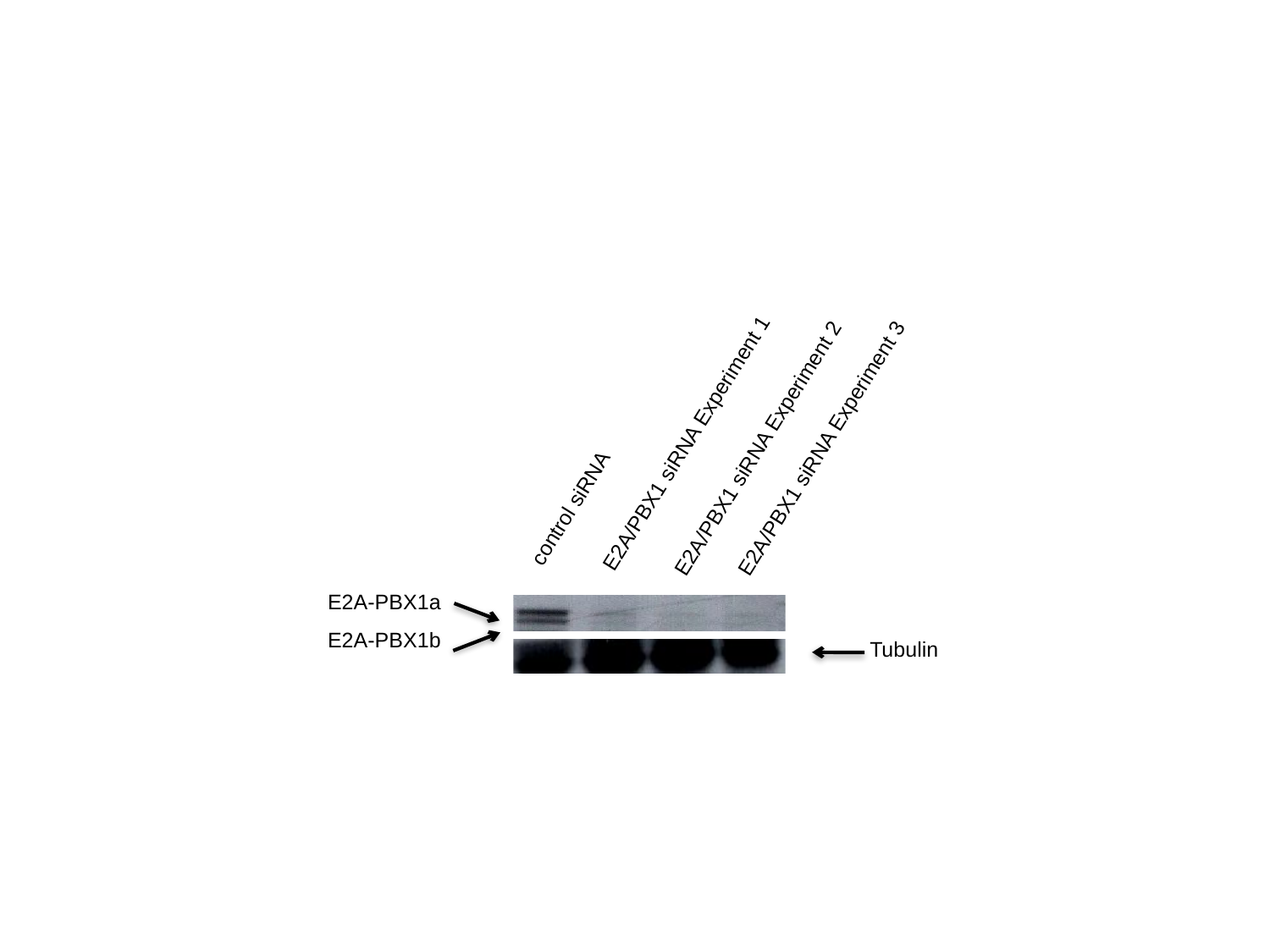

E2A/PBX1 siRNA Experiment 1
E2A/PBX1 siRNA Experiment 2
E2A/PBX1 siRNA Experiment 3
control siRNA
E2A-PBX1a
E2A-PBX1b
Tubulin
